# MicroRNA-20a Suppresses Tumor Proliferation and Metastasis in Hepatocellular Carcinoma by Directly Targeting EZH1

**DOI:** 10.3389/fonc.2021.737986

**Published:** 2021-12-16

**Authors:** Qianqian Zhang, Xiaohong Deng, Xiuxin Tang, Ying You, Meihua Mei, Danping Liu, Lian Gui, Yan Cai, Xiaoping Xin, Xiaoshun He, Junqi Huang

**Affiliations:** ^1^ Organ Transplant Center, The First Affiliated Hospital, Sun Yat-sen University, Guangzhou, China; ^2^ Guangdong Provincial Key Laboratory of Organ Donation and Transplant Immunology, The First Affiliated Hospital, Sun Yat-sen University, Guangzhou, China; ^3^ Guangdong Provincial Key Laboratory of Biomedical Imaging and Guangdong Provincial Engineering Research Center of Molecular Imaging, The Fifth Affiliated Hospital, Sun Yat-sen University, Zhuhai, China; ^4^ Guangdong Provincial International Cooperation Base of Science and Technology (Organ Transplantation), The First Affiliated Hospital, Sun Yat-sen University, Guangzhou, China; ^5^ Department of Laboratory Medicine, The First Affiliated Hospital, Sun Yat-sen University, Guangzhou, China

**Keywords:** hepatocellular carcinoma, microRNA-20a, EZH1, metastasis, epigenetics

## Abstract

**Purpose:**

Hepatocellular carcinoma (HCC), a worldwide leading cause of morbidity and mortality, is the most frequent primary liver tumor. Most HCC patients are diagnosed with advanced liver cancer, resulting in a very low 5-year survival rate. Thus, there is an urgent need for the development of targeted therapies. In this study, we aimed to investigate the effect and mechanism of the miR-20a/EZH1 axis on the proliferation and metastasis of HCC and the inhibitory effect of the EZH1/EZH2 inhibitor UNC1999 on HCC.

**Materials and Methods:**

The expression of miR-20a in human HCC tissues and cell lines was detected using quantitative real-time PCR (qRT-PCR). The expressions of proteins were analyzed with immunohistochemistry and Western blotting. Luciferase assay was used to verify whether miR-20a targets EZH1 or EZH2. The effect of miR-20a on HCC progression was studied *in vivo* and *in vitro*. The tumor inhibitory effect of UNC1999 was confirmed in vivo. CCK8 assay, wound healing assay, cell migration and invasion assay were used to evaluate the synergistic effect of UNC1999 with sorafenib. RNA sequencing (RNA-seq) was performed to screen the differentially expressed genes in the Huh7 and SMMC7721 cell lines after UNC1999, sorafenib, and combination treatments.

**Results:**

In this study, miR-20a showed a lower expression in both HCC tissues and cell lines. MiR-20a inhibited the proliferation and migration of SMMC7721 and Huh7 cells. The results of the luciferase assay and Western blot analysis revealed that miR-20a directly targeted EZH1, a histone methyltransferase. We demonstrated that miR-20a negatively regulated the expression of EZH1 and inhibited the proliferation and metastasis of HCC by reducing H3K27 methylation. We found UNC1999 inhibited tumor cells proliferation and enhanced the inhibitory effect of sorafenib.

**Conclusion:**

We demonstrated that miR-20a suppresses the tumor proliferation and metastasis in HCC by directly targeting EZH1. UNC1999 can inhibit tumor proliferation *in vivo* and increase the sensitivity of hepatoma cell lines to sorafenib.

## Introduction

Hepatocellular carcinoma (HCC), ranking seventh globally in incidence among malignant tumors, is characterized by a poor therapeutic effect and a high mortality rate, which makes it the third leading cause of cancer-related deaths in the world (1). Despite some advances in early detection and the recent improvements in treatment, the prognosis of patients with HCC remain poor. The current challenges are to identify new therapeutic targets and strategies and to incorporate these strategies into existing treatment regimens in order to improve treatment outcomes.

Over the past decade, mounting evidence implicates that microRNAs (miRNAs) have a well-recognized role in tumorigenesis (2–4). Currently, the understanding of the tumor-related role of miRNAs is divided into two main aspects: on one hand, the highly expressed miRNAs promote tumor progression by downregulating the expression of tumor suppressor genes; on the other hand, the low expression of miRNAs inhibits tumor formation by upregulating the expression of oncogenes (5). The altered expressions of several miRNAs (i.e., miR-18, miR-20a, miR-21, miR-34, miR-17-92, let-7a, let-7c, miR-92, miR-122, miR-195, miR-199a, miR-200a, miR-341, and miR-370) have been associated with HCC in mice and/or humans, but experimental evidence establishing a causal relationship between the abnormal expressions of these miRNAs and HCC is generally lacking (6). MiR-20a is one of the members of the miR-17-92 cluster that is located on chromosome 13 (13q31.3) (7). Downregulation of miR-20a was observed in primary HCC of patients following liver transplantation (6). Fan et al. reported that the decreased expression of miR-20a in HCC is related to its recurrence and prognosis (7), and new research has confirmed that miR-20a overexpression can inhibit liver cancer *in vivo* (8); however, the downstream mechanism of miR-20a remains unclear and merits further study.

Enhancer of zeste homologue 1 (EZH1) is a member of the Polycomb group (PcG) protein family that plays a crucial role in gene silencing (9, 10). Being one of the catalytic subunits of Polycomb repressive complex 2 (PRC2), EZH1 can repress the transcription of target genes by triggering the trimethylation of H3K27 (H3K27me3) (11). According to reports, EZH1 participates in the pathogenesis of many cancers (12–17), such as breast cancer, lung cancer, prostatic cancer, and hematologic malignancies.

However, the relationship between miR-20a and EZH1 and their functions in HCC remain undiscovered. Recent studies have revealed that several tumor suppressor miRNAs (i.e., miR-200c and miR-26a) are identified as the direct targets of EZH2 (18, 19). The role of EZH1 in HCC is far from clear. Exploring the miRNA/EZH1-associated signaling network in HCC development may provide novel therapeutic strategies for HCC treatment.

In this research, we identified that the expression of miR-20a is impaired in HCC tissues and cell lines, in a subcutaneous xenograft tumor model of HCC, due to EZH1-mediated epigenetic repression.

## Materials and Methods

### Patients and HCC Samples

Cancer and matched paracancerous tissues were obtained intraoperatively from 32 HCC patients in The First Affiliated Hospital, Sun Yat-sen University (Guangzhou, China), in 2015. Patients were included in this experiment upon giving informed consent. The present study met the approval of the Ethical Committee of the hospital. Details of the samples are summarized in **Table 1**.

**Table 1:** Clinicopathologic characteristics of HCC patients.

Feature	miR-20a: NO. of cases	EZH1: NO. of cases
Sex		
Female	2	3
Male	19	29
Age (years)		
<60	13	19
≥60	8	13
Tumor size (cm)		
<5	7	9
≥5	14	23
TNM stage		
I, II	11	16
III, IV	10	16
Portal vein tumor thrombus		
Yes	6	10
No	15	22
Cirrhosis		
Yes	6	5
No	16	27

### Reagents

UNC1999 and sorafenib were purchased from AbMole Bioscience (Houston, TX, USA). For cell culture experiments, UNC1999 was diluted in dimethyl sulfoxide (DMSO) to a stock of 10 mM, while sorafenib was diluted in DMSO to stocks of 100 µM.

### Cell Culture and Cell Transfection

In this study, we used human HCC cell lines, such as Huh7, Bel-7402, QGY-7703, SMMC7721, PLC8024, H2M, and H2P, and the human immortalized normal hepatocellular cell line MIHA, which were gifted by Professor Zhu Xiaofeng’s laboratory, Sun Yat-sen University Cancer Center. The HCC cells were cultured using Dulbecco’s modified Eagles’ medium (DMEM) (Gibco, Big Cabin, UK) supplemented with 10% fetal bovine serum (FBS), 100 μg/ml streptomycin, and 100 IU/ml penicillin at 37°C with 5% CO_2_. For cell transfection, the miRNA mimic, micrONTM mimic negative control (cy3), and siEZH1 were produced by RiboBio (Guangdong, China). The transfection buffer and reagent were also purchased from RiboBio. The miRNA mimic was transfected for 24 h and siEZH1 was transfected for 48 h in six-well plates with 3 × 10^5^ cells per well.

### Murine Subcutaneous Xenograft Tumor Model of HCC

Four-week-old male Balb/c nude mice were purchased from Nanjing Biomedical Research Institute of Nanjing University. A volume of 100 μl SMMC-7721 suspension cells, which contained 5 × 10^6^ cells, were injected subcutaneously into the flank region. In study of miR-20a, when the tumor reached a size of 5 mm × 5 mm, the mice were intratumorally injected with agomiR-20a or agomiR NC (both from RiboBio) twice a week. After 6 weeks, the gross morphology and tumor metastasis were assessed after euthanasia by macroscopically measuring the total size of the tumor nodules, conducted with hematoxylin and eosin (HE) staining of the tumor, liver, and lung. In the study of UNC1999, when the tumor reached a size of 100mm^3^, the mice were administered with DMSO as control or 150mg/kg UNC1999 once a day for 17 days. The tumor size was calculated with the following formula: volume (mm^3^) = [width^2^ × length]/2. All animal experiments were approved by the Ethics Committee for Clinical Research and Experimental Animals of the First Affiliated Hospital, Sun Yat-sen University.

### Quantitative Real-Time Polymerase Chain Reaction

RNA was extracted from cells and tissue samples with the Total RNA Kit (Omega Bio-Tek, Norcross, GA, USA) according to the manufacturer’s protocol. Reverse transcription was done with the Superscript III Kit (Invitrogen, Carlsbad, CA, USA). The expression level of mRNA was detected by quantitative real-time PCR (qRT-PCR) with an SYBR Green PCR Master Mix kit using a CFX96 System. The primers are shown in **Table 2**.

**Table 2:** Primers.

Primer	Sequence
GAPDH-F	GGGAAACTGTGGCGTGAT
GAPDH-R	GAGTGGGTGTCGCTGTTGA
EZH1-F	GCTTCCTTCACCCTTTTCATGCCACCC
EZH1-R	CGACGACCAGAGCACTTGGAG
EZH2-F	CCCTGACCTCTGTCTTACTTGTGGA
EZH2-R	ACGTCAGATGGTGCCAGCAATA
VEGFR-2-F	GTGATCGGAAATGACACTGGAG
VEGFR-2-R	CATGTTGGTCACTAACAGAAGCA
FGF21-F	GCCTTGAAGCCGGGAGTTATT
FGF21-R	GTGGAGCGATCCATACAGGG
SIRT1-F	AAGTTGACTGTGAAGCTGTACG
SIRT1-R	TGCTACTGGTCTTACTTTGAGGG
FGFR1-F	GGCTACAAGGTCCGTTATGCC
FGFR1-R	GATGCTGCCGTACTCATTCTC

### Cell Counting Kit-8 Assay

Cells were inoculated in a 96-well plate at 5 × 10^3^ cells/100 μl per well. Then, the cells were incubated with increasing concentrations of miR-20a (25, 50, and 100 μM), sorafenib, sorafenib combined with UNC1999 for 12, 24, or 48 h, respectively. The supernatant was removed and 10 µl Cell Counting Kit-8 (CCK-8) solution was added into each well containing 100 µl medium. After incubation for 3 h at 37°C, the absorbance of each group at 450 nm was detected (*n* = 3) using an absorbance microplate reader (Sunrise, Tecan, Grödingen, Austria).

### Colony Forming Assay

After miR-20a transfection, 500 cells were seeded into a six-well plate and allowed to grow for 10 days, during which period the medium was refreshed every 3 days. Then, the colonies were visualized with crystal violet staining.

### Cell Migration and Invasion Assay

After transfection with small interfering RNAs (siRNAs) or miRNAs or treated with sorafenib, and the combination of sorafenib and UNC1999, the cell concentration was adjusted to a value of 3 × 10^5^/ml in serum-free medium. In the migration assay, 200 μl of the suspended cells was plated into the upper chamber of the Transwell insert (Corning, Shanghai, China). Then, 600 μl culture medium with 15% FBS was added to the outside of the Transwell inserts. After 24 h, microscopic observations were performed. In the invasion assay, the Transwell inserts were treated with a Matrigel matrix (BD Biosciences, Franklin Lakes, NJ, USA) at 37°C for 2 h. Then, 200 μl of the suspended cells was seeded and incubated for 24 h. The rest of the procedures were similar to those in the migration assay.

### Wound Healing Assay for Migration

In the study of miR-20a, suspension cells (2 × 10^4^ cells/well) were inoculated into a 96-well plate for subsequent treatment and incubation. In the study of UNC1999, suspention cells (5 × 10^4^ cells/well) were incubated into a 6-well plate. Then, three parallel lines were slightly scratched. The cells were washed with PBS three times to remove the deciduous cells and incubated at 37°C for 24 h or 72 h.

### Western Blot Assay

SMMC7721 and Huh7 cells were lysed with RIPA (CWBio, Beijing, China) on ice. Cell protein lysates were separated by 10% SDS-PAGE, electrotransfered to polyvinylidene fluoride (PVDF) membrane (Roche, Basel, Switzerland), and blocked in 5% bovine serum albumin (BSA) for 1 h at room temperature. The membranes were incubated with primary antibody overnight at 4°C. The next day, the membranes were incubated with a secondary antibody for 1 h at room temperature. The bands were detected with an electrochemiluminescence (ECL) chromogenic substrate (Advansta, San Jose, CA, USA) and exposed using a fluorescence chemiluminescence imaging analysis system.

### Dual-Luciferase Reporter Assay

293T cells were plated into a 24-well plate for 24 h. The cells were then transfected with miRNA mimics and plasmids. After transfection for 48 h, the luciferase activities were measured with a dual-luciferase assay (Promega, Madison, WI, USA).

### Immunohistochemical Staining

The tumor tissues were deparaffinized, rehydrated, and blocked with 3% BSA. After blocking with BSA, the slices were incubated with primary antibodies against EZH1 (1:200), EZH2 (1:1,000), H3K27me (1:200), H3K27me2 (1:200), and H3K27me3 (1:500; all from Abcam, Cambridge, UK) overnight at 4°C. Then, the secondary antibody goat anti-rabbit horseradish peroxidase (HRP) was added and the slices incubated for 1 h at room temperature. A DAB reagent was added for the development of a brown color. Finally, the cell nucleases were stained with hematoxylin.

### *In Situ* Hybridization for miRNA

Paraffin-embedded tissue sections were cut and the paraffin removed. Then, the slices were placed in a boiling antigen repair reagent for antigen retrieval. Protease K was added to digest the slices for 15–25 min after cooling. After washing, the slices were incubated with a pre-hybridization reagent and then with a hybridization reagent. BSA was added to block the washed slices. Anti-digoxin-labeled alkaline phosphatase was added and incubated for 30 min after BSA was removed. The slices were then washed with Tris-buffered saline (TBS), after which 5-bromo-4-chloro-3-indolyl phosphate (BCIP)/nitro blue tetrazolium (NBT) was added. Finally, the cell nucleus was stained with a Nuclear Fast Red solution.

### RNA-Seq Analysis

SMMC7721 and Huh7 cells were treated with DMSO (control), 15 μM UNC1999 or sorafenib, or a combination of UNC1999 and sorafenib. RNA was extracted from the treated cells, and then the Illumina high-throughput sequencing platform was used for transcriptome sequencing. The edgeR algorithm was used to analyze the differentially expressed genes (DEGs) in each group after obtaining the sequence expression data. The Benjamini–Hochberg (BH) method was used to correct *p*-values for the multiple hypothesis test. Genes with *p* ≤ 0.05 and a fold-change (treated/untreated) cutoff >1 were considered as candidate DEGs. Both Gene Ontology (GO) annotation classification and Kyoto Encyclopedia of Genes and Genomes (KEGG) enrichment analysis were performed on the DEGs.

### Statistical Analysis

Each experiment was repeated at least three times. Quantitative data were presented as the mean ± SD. Statistical analysis was performed with SPSS 16.0 and GraphPad Prism 6.0 software. Student’s *t-*test and Pearson’s correlation analysis were adopted to evaluate statistical significance. A *p* < 0.05 was considered statistically significant.

## Results

### MiR-20a Expression Was Decreased in Human HCC Tissues

**Figure 1:**
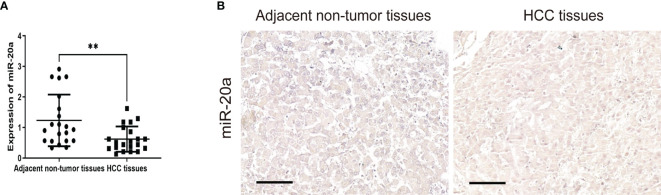
MiR-20a expression in human hepatocellular carcinoma (HCC) tissues. Quantitative real-time PCR (qRT-PCR) **(A)** and *in situ* hybridization **(B)** of miR-20a were used to detect the expression of miR-20a in HCC and its adjacent tissues. *Scale bar*, 100 μm (*n* = 21). **p < 0.01.

To explore the role of miR-20a in HCC, we collected tissue samples from HCC patients. qRT-PCR and miRNA *in situ* hybridization were performed to detect the expression of miR-20a in HCC and paratumorous tissues, which showed that the expression of miR-20a in HCC was significantly decreased compared with that in paratumorous tissues (**Figures 1A, B**).

### MiR-20a Expression and Its Inhibitory Function in the Proliferation and Metastasis of HCC Cells

**Figure 2:**
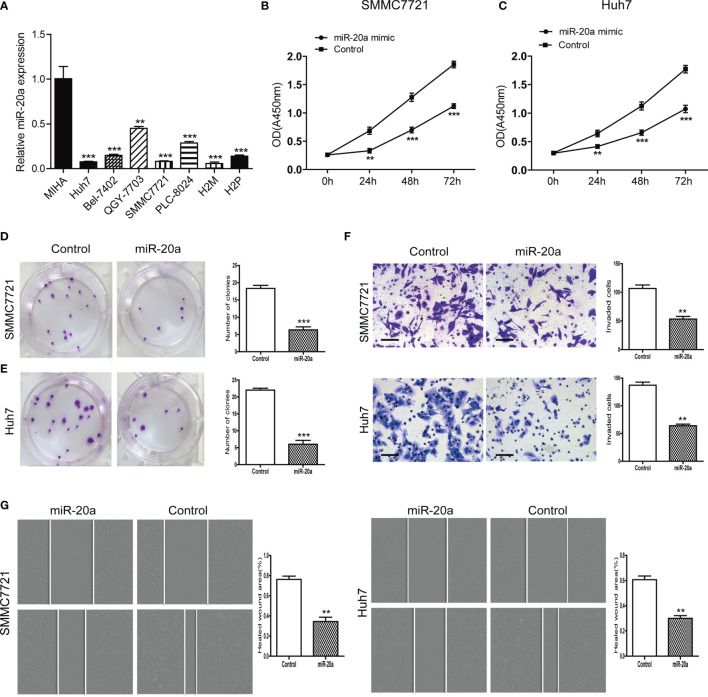
MiR-20a expression in hepatocellular carcinoma (HCC) cells and its inhibitory function in the proliferation and metastasis of HCC. **(A)** Expression level of miR-20a in seven HCC cell lines (MIHA as the normal liver cells) (*n* = 3). **p < 0.01, ***p < 0.001. **(B, C)** The proliferation of SMMC7721 and Huh7 cells was measured with the CCK-8 assay (*n* = 3). **p < 0.01, ***p < 0.001. **(D, E)** Colony formation assay in SMMC7721 and Huh7 cells transfected with miR-20a and its control. Representative pictures (*left*) and statistical data (*right*) are shown (*n* = 3). ***p < 0.001. **(F)** Cell invasion was analyzed using the Transwell system in SMMC7721 and Huh7 cells transfected with miR-20a and its control. *Scale bar*, 100 μm (*n* = 3). **p < 0.01. **(G)** Wound healing assay was used to establish the migration ability of the HCC cell lines SMMC7721 (*left*) and Huh7 (*right*) (*n* = 3). **p < 0.01.

We examined the expression levels of miR-20a in seven HCC cell lines (Huh7, Bel-7402, QGY-7703, SMMC7721, PLC-8024, H2M, and H2P) and in normal liver cells (MIHA). Compared with MIHA, the expression of miR-20a was lower in HCC cell lines, with a statistically significant difference (**Figure 2A**). Among them, SMMC7721 and Huh7 cells were the two strains with the lowest levels of miR-20a expression. Therefore, we selected SMMC7721 and Huh7 cells to conduct subsequent experiments in order to further study the function of miR-20a in HCC tumorigenesis. We transfected SMMC7721 and Huh7 cells with an miRNA mimic (miR-20a mimic) and found that the expression of miR-20a significantly increased (**Supplementary Figures S1** and **S2**). The results of the CCK-8 and colony formation assays revealed that there was a reduction in cell proliferation after transfection with miR-20a (**Figures 2B–E**). Subsequently, we found that, after transfection for 24 h, there were fewer cells invading the other side of the Transwell membrane (**Figure 2F**). Moreover, the healed wound area was smaller in the miR-20a transfection group, showing that miR-20a inhibited the migration capacity of SMMC7721 and Huh7 (**Figure 2G**). Therefore, miR-20a was downregulated in the HCC cell lines, and an upregulated miR-20a inhibited the proliferation and metastasis of the HCC cell lines.

### MiR-20a Directly Targeted EZH1

**Figure 3:**
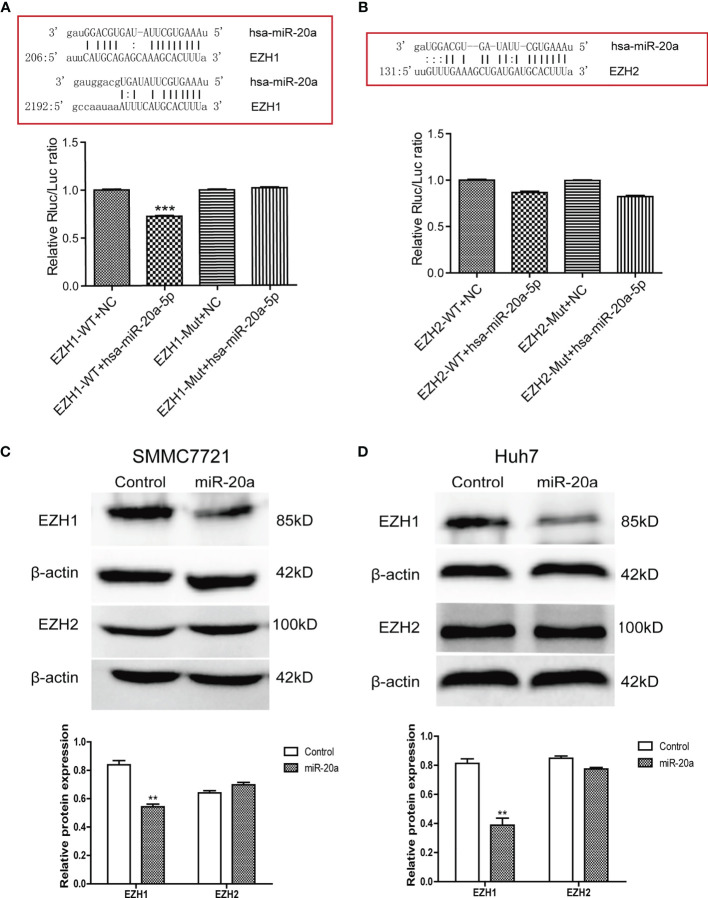
MiR-20a directly targeted EZH1. **(A, B)** Bioinformatics analysis and luciferase assay were performed to detect the interaction of miR-20a with EZH1 and EZH2 (*n* = 3). ***p < 0.001. **(C, D)** After transfection of miR-20a and its control, the protein levels of EZH1 and EZH2 in SMMC7721 and Huh7 cells were detected using specific antibodies (*n* = 3). **p < 0.01.

To explore the potential regulatory mechanisms of miR-20a in HCC migration and proliferation, we analyzed the target genes associated with miR-20a using the software TargetScan (http://www.targetscan.org/vert_71/), miRWalk (http://mirwalk.umm.uni-heidelberg.de/), and miRDB (http://mirdb.org/). The results of the bioinformatics analysis showed that miR-20a may target the histone methyltransferase, the enhancer of zeste homologs 1 and 2 (EZH1 and EZH2). To verify the direct binding of miR-20a to the 3′UTR of EZH1/EZH2, we used the dual-luciferase reporter assay. The results showed that miR-20a could directly target EZH1, but not EZH2. MiR-20a overexpression markedly decreased the luciferase activities in the EZH1 wild-type (WT) group, but not in the EZH1 mutant group, indicating that miR-20a directly targeted the 3′UTR of EZH1 (**Figure 3A**). However, there was no direct binding between miR-20a and EZH2 (**Figure 3B**). Similarly, the inhibitory ability of miR-20a in the expression of the EZH1 protein was verified by Western blotting (**Figures 3C, D**). The results suggested that *EZH1* is a direct target gene of miR-20a in HCC.

### Expression of EZH1 and H3K27 Methylation in Human HCC Tissues

**Figure 4:**
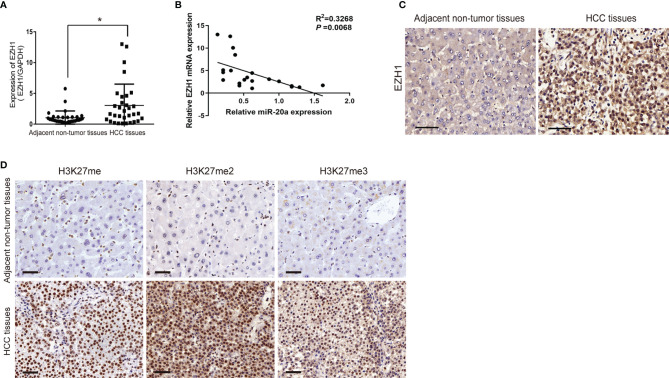
Expression of EZH1 in human hepatocellular carcinoma (HCC) tissues. **(A, C)** Quantitative real-time PCR (qRT-PCR) and immunohistochemistry were employed to measure the expression of EZH1 in hepatocellular carcinoma and its adjacent tissues. *Scale bar*, 50 μm (*n* = 32). *p < 0.05. **(B)** An inverse correlation between the expression levels of miR-20a and EZH1 was obvious in HCC tissues. *p*=0.0068. **(D)** The expressions of H3K27me, H3K27me2, and H3K27me3 in HCC and its adjacent tissues were detected by immunohistochemistry. *Scale bar*, 50 μm.

EZH1 expression was significantly increased in HCC tissues (**Figure 4A**). EZH1 was positively expressed in HCC, but negatively expressed in paratumorous tissues (**Figure 4C**). Thus, there was a negative correlation between miR-20a and EZH1 in HCC tissues (**Figure 4B**). MiR-20a might affect HCC progression by regulating EZH1, which is one of the core members of PRC2 and has histone methyltransferase activity. Using immunohistochemistry, we found that histone H3 lysine 27 methylation (H3K27me) occurred in HCC tissues and that the expressions of H3K27me, H3K27me2, and H3K27me3 were upregulated (**Figure 4D**). Therefore, EZH1 might inhibit the target gene expression through modifications in H3K27 methylation, eventually leading to tumorigenesis.

### Knockdown of EZH1 Inhibited HCC Proliferation and Metastasis

**Figure 5:**
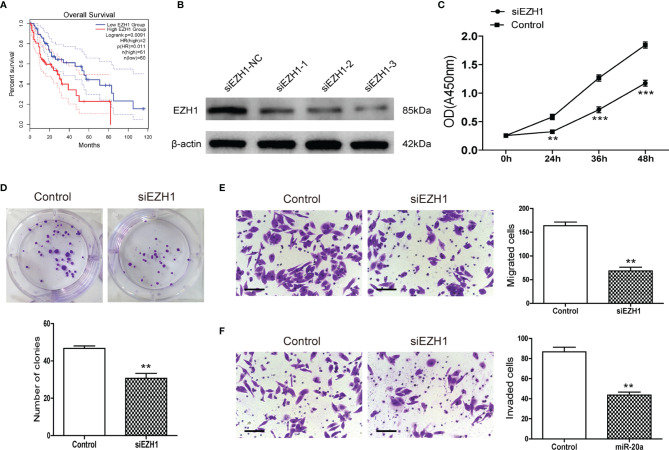
Knockdown of EZH1 inhibited the proliferation and metastasis of hepatocellular carcinoma (HCC). **(A)** Kaplan–Meier survival curves show the overall survival (OS) rate of HCC patients with high (*n* = 180) and low (*n* = 181) protein expression levels of EZH1 from the GEPIA database. **(B)** Transfection of siEZH1 in the HCC cell line SMMC7721 inhibited the expression of EZH1. **(C)** The proliferation of SMMC7721 was measured using the CCK-8 assay (*n* = 3). **p < 0.01, ***p < 0.001. **(D)** Colony formation assay in SMMC7721 transfected with siEZH1 and its control. Representative pictures (*top*) and statistical data (*bottom*) are shown (*n* = 3). **p < 0.01. **(E)** Transwell assay analysis of SMMC7721 was performed after transfection with siEZH1 and its control to determine the migration capacity. *Scale bar*, 100 μm (*n* = 3). **p < 0.01. **(F)** Transwell assay analysis of SMMC7721 was performed after transfection with siEZH1 and its control to determine the invasion capacity. *Scale bar*, 100 μm (*n* = 3). **p < 0.01.

MiR-20a could directly regulate EZH1, but the function of EZH1 in HCC is unclear. We used the Kaplan–Meier plot (20) to determine the prognostic value of EZH1 in HCC, with the results indicating that patients with a low EZH1 expression show better overall survival (OS) compared to those with a high EZH1 expression (*p*< 0.05) (**Figure 5A**). To further elucidate the role of EZH1, SMMC7721 cells were transfected with three types of siEZH1 for 48 h to confirm the reduction of EZH1 expression. The results of Western blot analysis revealed that the expression level of EZH1 was significantly decreased (**Figure 5B**). Therefore, we selected siEZH1-1 for further experiments. The results of both the CCK-8 assay (**Figure 5C**) and the colony formation assay (**Figure 5D**) showed that the proliferation ability of SMMC7721 was markedly inhibited in the siEZH1-1 group. Moreover, siEZH1 also significantly suppressed the migration and invasion ability of SMMC7721 (**Figures 5E, F**).

### MiR-20a Inhibited HCC Formation and Metastasis by Directly Targeting EZH1 *In Vivo*

**Figure 6:**
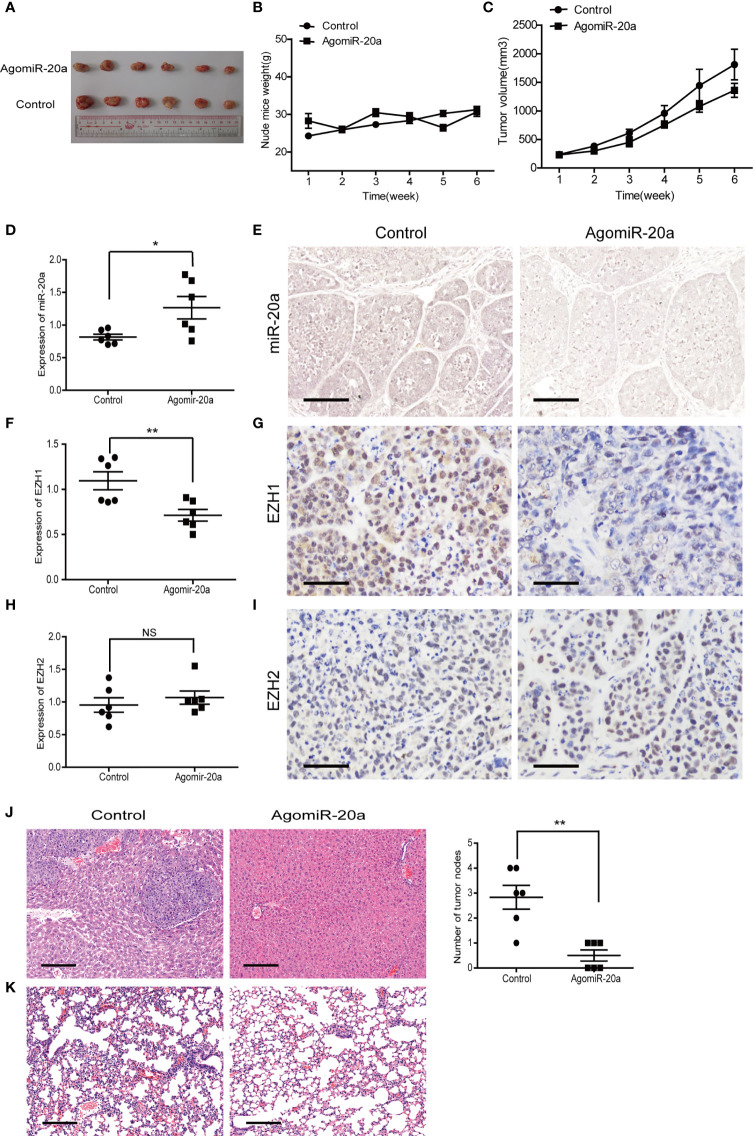
MiR-20a inhibited tumorigenicity *in vivo*. **(A–C)** Tumors formed in nude mice. SMMC7721 cells were injected into the flanks of nude mice by subcutaneous injection. Mice were killed after 6 weeks. Representative pictures of solid tumors **(A)**, tumor weight **(B)**, and tumor volume **(C)** are presented (*n* = 6). **(D, F, H)** Expressions of miR-20a **(D)**, EZH1 **(F)**, and EZH2 **(H)** in subcutaneous tumors of nude mice detected by quantitative real-time PCR (qRT-PCR) (*n* = 6). *p < 0.05, **p < 0.01, (NS denotes the absence of significant difference). **(E)** MicroRNA *in situ* hybridization was employed to detect the expression of miR-20a in subcutaneous tumors of nude mice. *Scale bar*, 100 μm. **(G)** EZH1 expression in subcutaneous tumors of nude mice detected by immunohistochemistry. EZH1 expression in the nucleus: positive expression is shown in *brown* and negative in *blue*. *Scale bar*, 50 μm. **(I)** EZH2 expression in subcutaneous tumors of nude mice detected by immunohistochemistry. EZH2 expressed in the nucleus: positive expression is shown in *brown* and negative in *blue*. *Scale bar*, 50 μm. **(J, K)** Liver and lung metastases of the miR-20a agonist group and its control shown using hematoxylin–eosin (HE). *Scale bar*, 200 μm.

To determine the effect of miR-20a on HCC tumorigenicity, we performed tumorigenesis experiments in nude mice. SMMC7721 cells were subcutaneously injected into nude mice. After tumor formation (more than 5 mm × 5 mm), the mice were divided into two groups. The miR-20a agonist (agomiR-20a) and its control were continuously injected for 6 weeks. The tumor growth inhibitory rate and the tumor volume decrease rate were calculated. There was no significant change in the body weight of the mice in the two groups (**Figure 6B**). However, the tumor volume in the mice of the agomiR-20a group decreased to some degree (**Figure 6C**). These results indicated that miR-20a inhibited the growth of HCC. Subsequently, we examined the expressions of miR-20a, EZH1, and EZH2 in nude mice using qRT-PCR, *in situ* hybridization, and immunohistochemistry. Compared with that in the control group, the expression of miR-20a increased in the agomiR-20a group (*p* < 0.05), whereas that of EZH1 decreased (*p* < 0.01), the expression of EZH2 showed no significant change (*p* > 0.05) (**Figures 6D–I**). Then, we dissected the mice and detected visible liver metastasis in the control group. The HE staining results clearly showed the presence of tumor cells; the structure of the hepatic lobules disappeared and an obvious necrosis of the liver cells around the tumor was observed. However, there was no liver metastasis in the agomiR-20a group (**Figure 6J**). An increased number of cells was found in the lungs of nude mice, as well as a large number of infiltrated inflammatory cells in the control group, but not in the agomiR-20a group (**Figure 6K**).

### UNC1999, an Oral EZH1/EZH2 Inhibitor, Inhibited HCC Formation *In Vivo*

**Figure 7:**
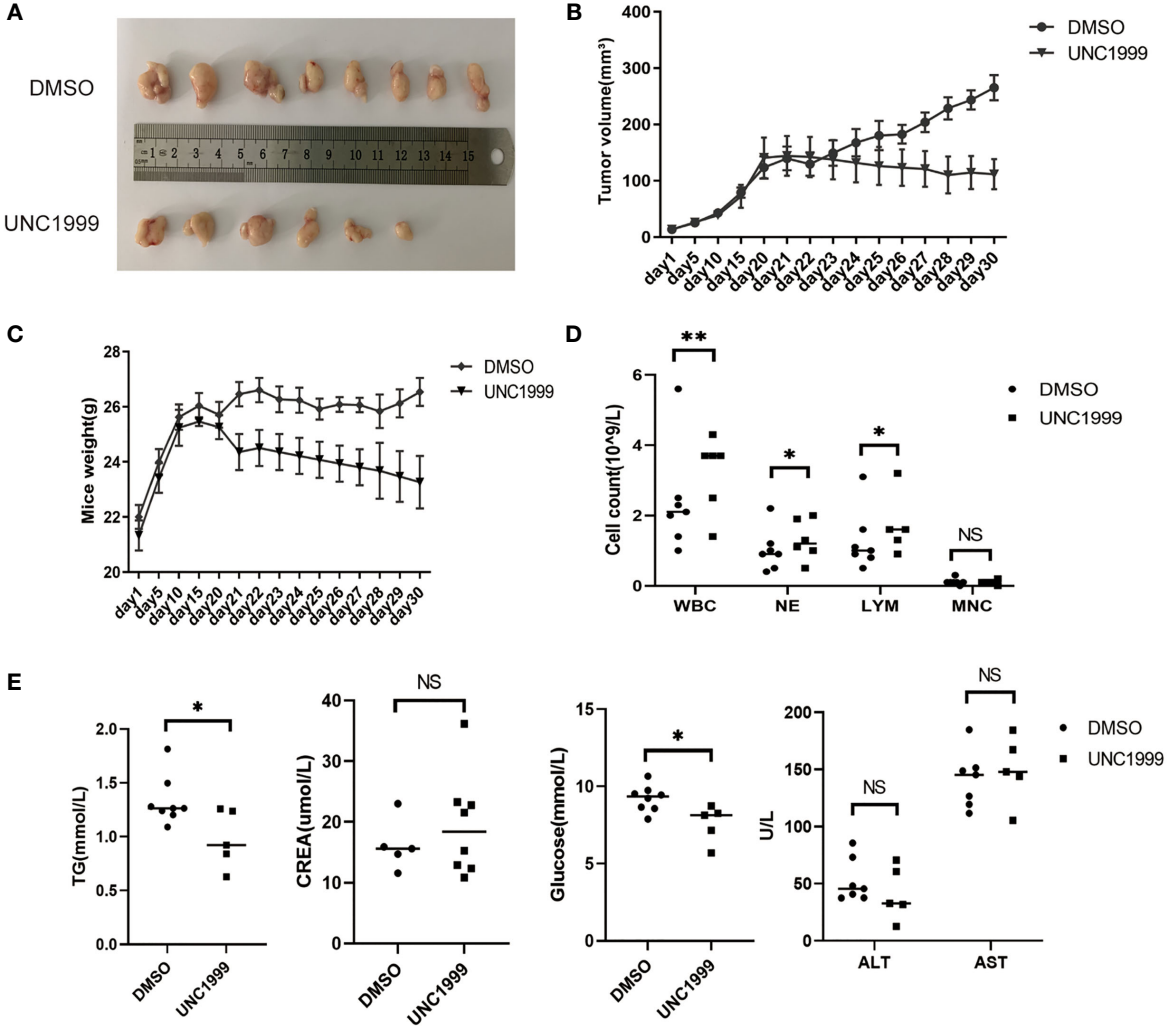
UNC1999 inhibited tumorigenicity *in vivo*. **(A–C)** Tumors formed in nude mice. SMMC7721 cells were injected into nude mice by subcutaneous injection. On day 20 after injection, mice with tumor were randomly divided into two groups and were given DMSO as control or UNC1999 (150mg/kg) separately by gavage once a day. After 17 days, mice were euthanized. **(A)** Representative pictures of solid tumors (DMSO group n=8, UNC1999 group n=6). **(B)** Tumor volume. **(C)** Mice weight. **(D)** The numbers of white blood cells (WBC), neutrophils (NE), lymphocytes (LYM) and monocytes (MNC) in peripheral blood of mice in DMSO group and UNC1999 group. *p < 0.05, **p < 0.01, NS denotes the absence of significant difference. **(E)** The levels of triglyceride (TG), creatinine (CREA), glucose, ALT and AST in plasma of mice in DMSO group and UNC1999 group. (*p < 0.05, NS denotes the absence of significant difference).

UNC1999 was developed as the first oral EZH1/EZH2 inhibitor in 2013 (21). We have found that UNC1999 had inhibitory effect in HCC cells in our previous study (22). We used murine subcutaneous xenograft tumor model to evaluate the curative effect and safety of UNC1999 in HCC. There were no statistical significant changes in body weight and the tumor volume in the two groups (**Figures 7A–C**).

However, they decreased to some degree in the UNC1999 group. The number of white blood cells (WBC), neutrophils (NE), lymphocytes (LYM) and monocytes (MNC) in peripheral blood was higher in the UNC1999 group (**Figure 7D**), but still in the normal reference range. There was no significant difference in plasma creatinine (CREA), alanine aminotransferase (ALT) and aspartate aminotransferase (AST) (**Figure 7E**) between the two groups, suggesting that UNC1999 had a very finite liver and renal toxicity. Blood glucose and triglycerides were significantly lower in UNC1999 group (**Figure 7E**), which suggests that UNC1999 may affect glucose and lipid metabolism.

### UNC1999 Enhanced the Inhibitory Effects of Sorafenib in HCC Cells

**Figure 8:**
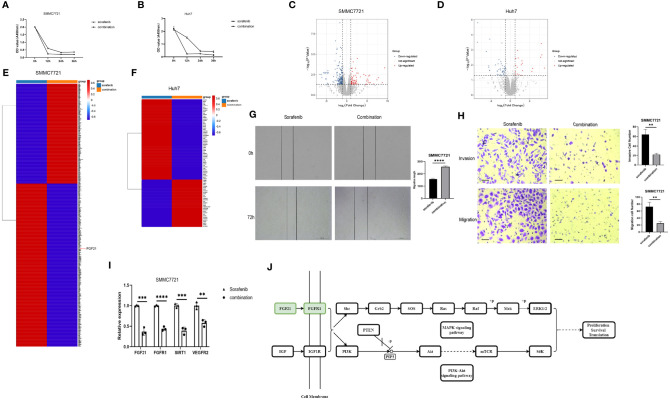
UNC1999 enhanced sorafenib inhibitory effects in hepatocellular carcinoma cell lines. **(A, B)** Proliferation of SMMC7721 **(A)** and Huh7 **(B)** was measured by using CCK8 assay. **(C, D)** Volcano plots of DEGs between sorafenib and combination group of SMMC7721 **(C)** and Huh7 **(D)**. **(E, F)** Expression of up-regulated and downregulated DEGs that were related to HCC proliferation and metastasis of sorafenib group and combination group in SMMC7721 **(E)** and Huh7 **(F)** were presented in heatmaps. **(G)** Wound healing assay was used to establish the migration ability of SMMC7721 (n=3, ****P < 0.0001). **(H)** Cell invasion and migration in SMMC7721 were analyzed using the Transwell assay. Scale bar, 500μm (n=3, **P < 0.01). **(I)** The expression of FGF21, FGFR1, SIRT1 and VEGFR2 in SMMC7721 was measured by qRTPCR. **p < 0.01, ***p < 0.001, ****p < 0.0001. **(J)** Diagram of FGF21-related KEGG pathway. sorafenib group: 15μM sorafenib; combination group: 15μM sorafenib and 15μM UNC1999.

**Figure 9:**
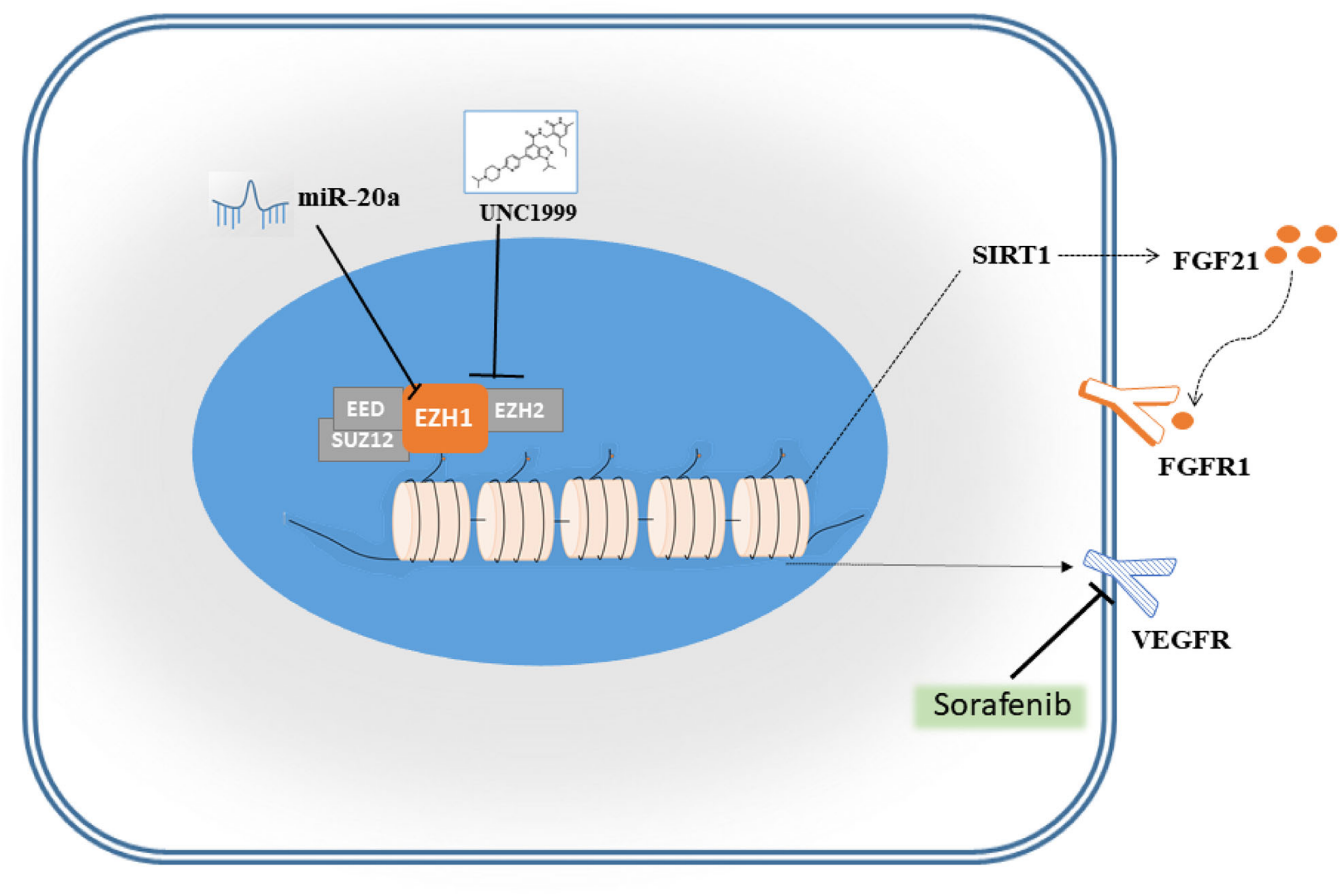
Schematic diagram of miR-20a and EZH1.

Sorafenib is a tyrosine kinase inhibitor that shows antitumour effects against various cancers (23). However, the clinical application of sorafenib is often hampered by drug resistance (23). To explore synergistic effect of UNC1999 with sorafenib in HCC, we used sorafenib and combination of sorafenib and UNC1999 in SMMC7721 and Huh7 cells. CCK8 assay results showed that the optimal dosage was 15 µM for 12 h (**Figures 8A, B**). RNA-seq analysis was performed to seek out DEGs in SMMC7721 and Huh7 cells. We identified up-regulated and down-regulated DEGs by using volcano plots (**Figures 8C, D**). Heatmap of DEGs revealed the related genes may have synergistic effect in HCC cells (**Figures 8E, F**). Results of wound healing assay and Transwell experiment showed that UNC1999 significantly enhanced sorafenib inhibitory ability in HCC cells (**Figures 8G, H**). Bioinformatics (RNA-seq) analysis indicated that, compared with sorafenib group, the transcription level of *FGF21* in the combination group decreased significantly and was verified by qRT-PCR. FGF21 can promote cell proliferation, vascular proliferation, and wound repair. It participates in maintaining the balance of energy metabolism and is closely related to liver diseases. In patients with liver tumors, the levels of FGF21 in hepatocytes were invariably elevated compared to those in healthy controls (24). Therefore, we used qRT-PCR to verify the receptor of FGF21 (*FGFR1*), the targets of sorafenib (*VEGFR2*) (23) and upstream gene of FGF21 (*SIRT1*). These genes were all down-regulated in the combination group (**Figure 8I**). We found a pathway related to FGF21 (**Figure 8J**) in KEGG website (https://www.kegg.jp/), which showed FGF21-FGFR1 binding targeted the cell proliferation, cell survival and translation via PI3K-Akt and MAPK signaling pathways. The plasma level of FGF21 was reduced in sorafenib-treated HCC patients (25), which suggested that FGF21 might be related with sorafenib treatment in HCC. Finally, we took a diagram to show our work (**Figure 9**), miR-20a and UNC1999 inhibited tumor proliferation and metastasis by inhibiting EZH1. At the same time, UNC1999 could increase the sensitivity of hepatoma cell lines to sorafenib by down regulating *SIRT1*, *FGF21*, *FGF21* and *VEGFR2*.

## Discussion

In the current study, we found that miR-20a was suppressed in HCC tissues (**Figures 1A, B**) and that its overexpression inhibited the growth of HCC cells, as shown by the results of the CCK-8 and colony formation assays (**Figures 2A–E**). We speculated that miR-20a played an inhibitory regulation role in HCC and that its low expression could accelerate the development of HCC. The results of the Transwell and wound healing assays of SMMC7721 and Huh7 cells showed that miR-20a inhibited the invasion and migration of HCC (**Figures 2F, G**). In addition, we also verified our findings in animals. With animal studies *in vivo*, we observed that there were no significant changes in the tumor size and body weight of the animals in the two groups (**Figures 6A–C**). Interestingly, there was obvious tumor metastasis in the liver of mice in the control group. However, the miR-20a overexpression group showed no appearance of metastases (**Figure 6J**). Inflammation was detected in the lungs of the control group of animals, which was absent in the miR-20a group (**Figure 6K**). In summary, we found that miR-20a overexpression inhibited HCC cell proliferation, invasion, and migration *in vitro* and *in vivo*.

MiR-20a is critically involved in the proliferation and metastasis of HCC cells, but its specific regulatory mechanism has not been elucidated yet. Based on these results and the data from the bioinformatics analysis, we speculated that miR-20a played indirect regulatory functions in the pathogenesis of HCC. An earlier study revealed that miR-17-5p inhibited EZH1 and that the downregulation of miR-17-5p was associated with drug resistance in lung cancer cells by targeting EZH1 (26). Through bioinformatics analysis and luciferase assays, we discovered that miR-20a, which is located in the same gene cluster as miR-17-5p, can directly target EZH1, but not EZH2, an EZH1 homolog (**Figures 3A, B**). The Western blot results further validated our speculation that the overexpression of miR-20a inhibited the protein levels of EZH1 (**Figures 3C, D**). EZH1 is a vital member of the polyclonal family PcG, which can elicit epigenetic silencing by regulating the remodeling of chromatin. The PcG gene forms two Polycomb inhibitory complexes (PRC1 and PRC2) by complex coding different proteins. The PRC1 complex consists of CBX, PHC, BMI1, and RING1A, and RING1B (27). On the other hand, the PRC2 complex consists of EZH1, EZH2, SUZ12, EED, RBBP2, and AEBP2 (28). EZH1 is an important catalytic subunit of the PRC2 complex. EZH1 could methylate lysine 27 of histone H3 methylation, and the PRC1 complex recognizes and binds to specific gene loci, leading to the repression of gene transcription (29, 30). Currently, the high expression of EZH1 in some tumors, such as in lung cancer, breast cancer, and prostate cancer, suggests that it is closely related to tumorigenesis. Our group found that the expression of EZH1 in HCC was significantly upregulated (**Figures 4A, C**). We also established that the expressions of H3K27me, H3K27me2, and H3K27me3 were upregulated in HCC patients (**Figure 4D**), indicating that EZH1 may participate in H3K27 methylation, causing cell proliferation and gene transcription inhibition and ultimately leading to the occurrence of tumors.

Our results showed that EZH1/2 inhibitor UNC1999 had a tendency to inhibit the prolifeation of liver cancer *in vivo*, then we performed RNA-seq on the SMMC7721 and Huh7 cell lines treated with sorafenib and a combination of sorafenib and UNC1999. UNC1999 is an orally bioavailable selective dual EZH1/EZH2 inhibitor that works in competition with the cofactor *S*-adenosyl-l-methionine (SAM) (21). UNC1999 has shown inhibitory effects on a variety of tumors, such as mixed lineage leukemia (MLL), rearranged leukemia, and multiple myeloma (31). Sorafenib is an orally bioavailable multi-kinase inhibitor that was approved by the Food and Drug Administration (FDA) for clinical use in the treatment of HCC in 2007 and can block tumor angiogenesis by inhibiting VEGFR and platelet-derived growth factor receptor (PDGFR) (32). Many patients treated with sorafenib eventually developed resistance to the drug. Our results showed that UNC1999 could reduce the expression of VEGFR-2 and achieve maximum inhibition in combination with sorafenib. Therefore, VEGFR-2 is one of the effective targets of UNC1999. On the other hand, in the sorafenib group and the combination group, FGF21, one of the fibroblast growth factors, and its relative genes *FGFRl* and *SIRT1* were down-regulated. Studies (33, 34) have shown that FGF21 is an important activator of the PI3K signaling pathway, one of the signaling pathways in initiating and promoting HCC. Our KEGG analysis results showed that the PI3K/Akt signaling pathway was significantly down-regulated, which exactly confirmed that the target of UNC1999 may be the FGF21/PI3K/Akt signaling pathway. Consequently, VEGFR-2, FGF21, and its downstream PI3K/Akt signaling pathway may explain the inhibition and synergistic effects of UNC1999.

The present study has several limitations. Firstly, we did not have enough tissue samples from HCC patients to examine the expressions of miR-20a and EZH1. Secondly, the results of the RNA-seq analysis were not validated using qRT-PCR and Western blot assay globally. Finally, further in-depth analysis is required to study the roles of miR-20a and EZH1 in HCC tumorigenesis and to explore miR-20a as a potential prognostic target.

## Conclusion

In summary, miR-20a negatively regulates the expression of EZH1 and reduces the methylation of H3K27. The EZH1/EZH2 inhibitor UNC1999 has inhibitory effects on the metastasis, invasion, and migration of HCC. However, the specific role of EZH1 in HCC remains to be further studied.

## Data Availability Statement

The datasets presented in this study can be found in online repositories. The names of the repository/repositories and accession number(s) can be found below: http://www.ncbi.nlm.nih.gov/bioproject/765866.

## Ethics Statement

The studies involving human participants were reviewed and approved by the Ethics Committee for Clinical Research and Experimental Animals of the First Affiliated Hospital, Sun Yat-sen University. The patients/participants provided written informed consent to participate in this study. The animal study was reviewed and approved by the Ethics Committee for Clinical Research and Experimental Animals of the First Affiliated Hospital, Sun Yat-sen University.

## Author Contributions

JH conceived and designed the experiments. QZ and XD performed research, analyzed the data, and wrote the paper. XT, YY, MM, and DL performed research, analyzed the data, and wrote part of the paper related to EZH1 inhibitor UNC1999 function. LG, YC, and XX analyzed the results and improved the manuscript. XH provided clinical samples. All authors supervised the research, analyzed the results, and improved the manuscript. All authors contributed to the article and approved the submitted version.

## Funding

This work was supported by the National Natural Science Foundation of China (NO. 31370870 and NO. 81871238), the Natural Science Foundation of Guangdong Province (NO. S2013020013000 and NO. 2021A1515010435), the Science and Technology Program of Guangdong, China (NO. 2013A020229003, NO. 2015B050501002, NO. 2020A0505020003, and NO. 2020B1212060026), and the Science and Technology Program of Guangzhou, China (NO. 201604020083 and no. 202103000007).

## Conflict of Interest

The authors declare that the research was conducted in the absence of any commercial or financial relationships that could be construed as a potential conflict of interest.

## Publisher’s Note

All claims expressed in this article are solely those of the authors and do not necessarily represent those of their affiliated organizations, or those of the publisher, the editors and the reviewers. Any product that may be evaluated in this article, or claim that may be made by its manufacturer, is not guaranteed or endorsed by the publisher.

## Supplementary Material

Supplementary Figure 1 Cy3-miRNA mimic NC can effectively transfect hepatocellular carcinoma cells SMMC7721. **(A)** Cy3-labeled mimic NC was observed under a fluorescence microscope to preliminary assess transfection efficiency in SMMC7721. **(B)** The expression of miR-20a in SMMC7721 hepatocellular carcinoma cells was detected by qRT-PCR 24h, 36h and 48h after transfection. (MiR-20a mimic *vs* miR-20a mimic NC, *n* = 3, ****p* < 0.001, ***p* < 0.01).

Supplementary Figure 2 Cy3-miRNA mimic NC can effectively transfect liver cancer cells Huh7. **(A)** Cy3-labeled mimic NCs were observed under a fluorescence microscope to initially assess transfection efficiency in Huh7. **(B)** The expression of miR-20a in Huh7 cells was detected by qRT-PCR 24h, 36h and 48h after transfection. (MiR-20a mimic *vs* miR-20a mimic NC, n = 3, ****p* < 0.001).
