# Supplementary figures and images for: MicroRNA-20a Suppresses Tumor Proliferation and Metastasis in Hepatocellular Carcinoma by Directly Targeting EZH1
Source: Front Oncol. 2021 Dec 16;11:737986. doi: 10.3389/fonc.2021.737986 (PMC8716374; doi:10.3389/fonc.2021.737986)

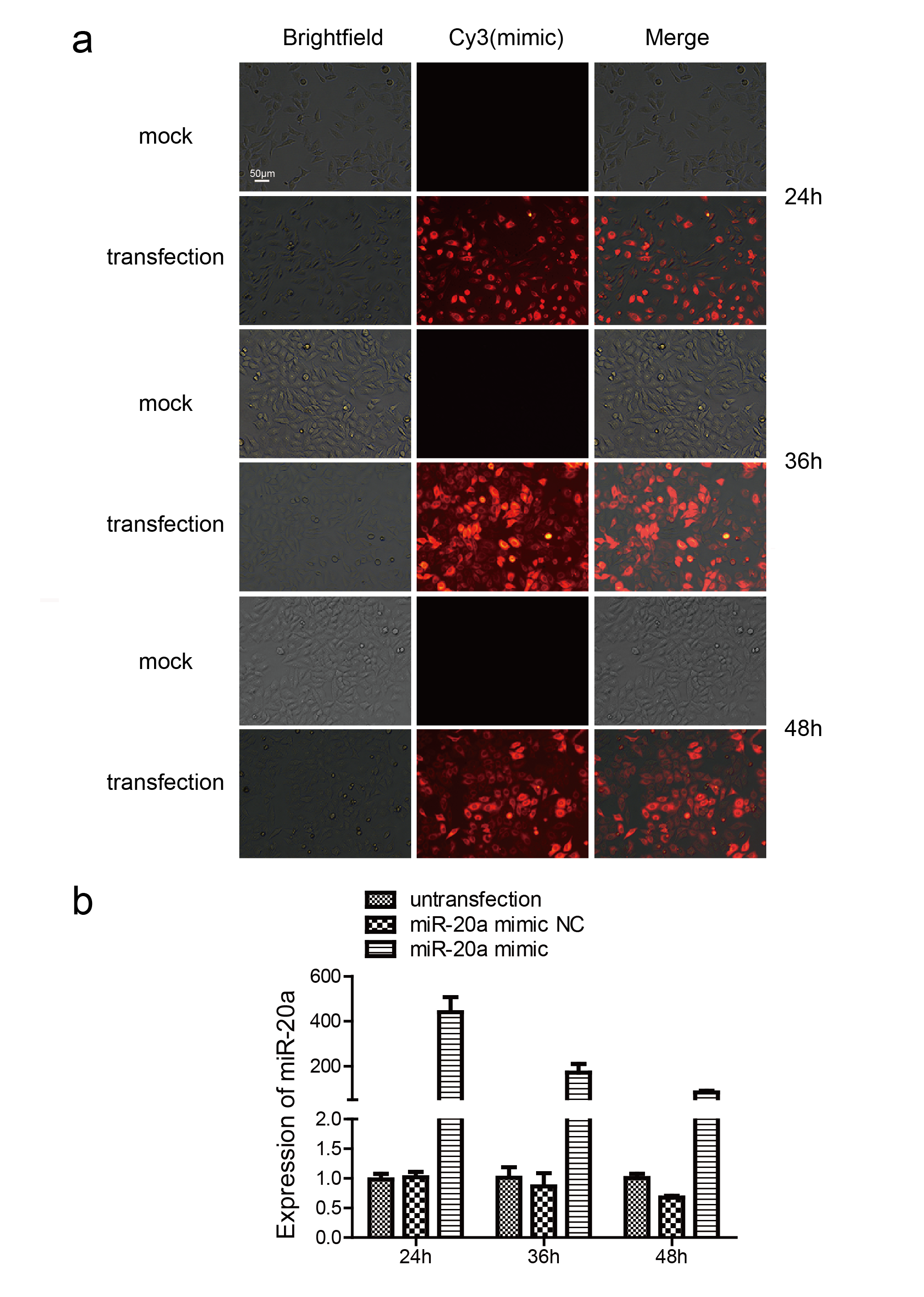

Supplement: Supplementary Figure 1 — Cy3-miRNA mimic NC can effectively transfect hepatocellular carcinoma cells SMMC7721. (A) Cy3-labeled mimic NC was observed under a fluorescence microscope to preliminary assess transfection efficiency in SMMC7721. (B) The expression of miR-20a in SMMC7721 hepatocellular carcinoma cells was detected by qRT-PCR 24h, 36h and 48h after transfection. (MiR-20a mimic vs miR-20a mimic NC, n = 3, ***p < 0.001, **p < 0.01). [file Image_1.tif]

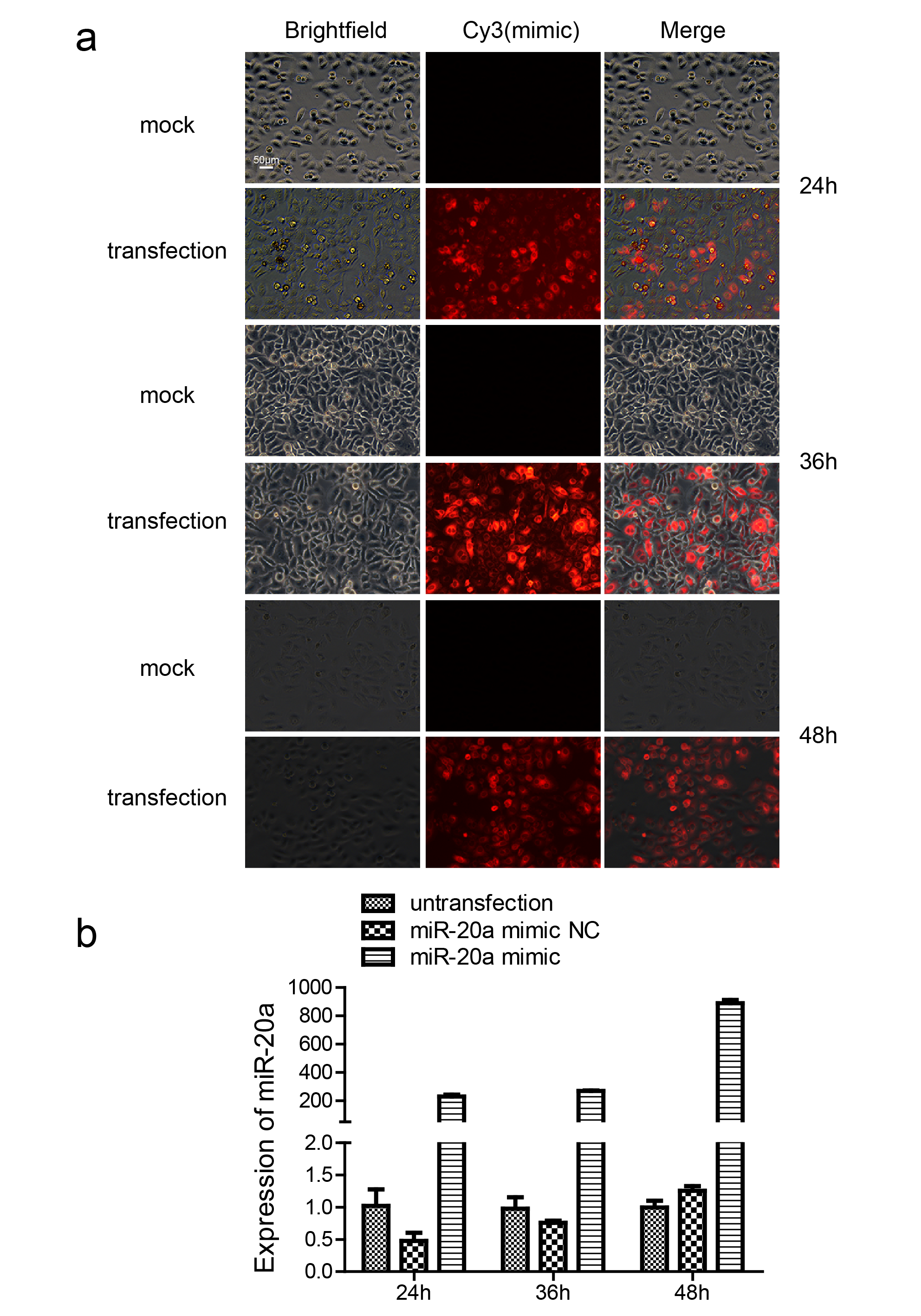

Supplement: Supplementary Figure 2 — Cy3-miRNA mimic NC can effectively transfect liver cancer cells Huh7. (A) Cy3-labeled mimic NCs were observed under a fluorescence microscope to initially assess transfection efficiency in Huh7. (B) The expression of miR-20a in Huh7 cells was detected by qRT-PCR 24h, 36h and 48h after transfection. (MiR-20a mimic vs miR-20a mimic NC, n = 3, ***p < 0.001). [file Image_2.tif]
